# Cooperative Recruitment of FtsW to the Division Site of *Bacillus subtilis*

**DOI:** 10.3389/fmicb.2016.01808

**Published:** 2016-11-15

**Authors:** Pamela Gamba, Leendert W. Hamoen, Richard A. Daniel

**Affiliations:** Centre for Bacterial Cell Biology, Institute for Cell and Molecular Biosciences, Newcastle UniversityNewcastle upon Tyne, UK

**Keywords:** FtsZ, FtsW, FtsL, cell division, *Bacillus subtilis*

## Abstract

Five essential proteins are known to assemble at the division site of *Bacillus subtilis*. However, the recruitment of the FtsW homolog is still unclear. Here, we take advantage of spore germination to facilitate the depletion of essential proteins and to study the divisome assembly in the absence of previous division events. We show that, unlike what has been shown for the *Escherichia coli* divisome, the assembly of FtsW is interdependent with the localization of PBP 2B and FtsL, which are key components of the membrane bound division complex. Interestingly, the Z-ring appeared to disassemble upon prolonged depletion of late division proteins. Nevertheless, we could restore Z-ring formation and constriction by re-inducing FtsW, which suggests that the stability of the Z-ring is stimulated by the assembly of a functional division complex.

## Introduction

The division of a bacterial cell requires the coordinated synthesis of new cell membrane and cell wall, and is achieved by a dynamic protein complex known as the divisome (Nanninga, 1991; Errington et al., 2003; Adams and Errington, 2009). Cell division is initiated by the assembly of the protein FtsZ into a ring-like structure at midcell. FtsZ assembles concomitantly with FtsA, which anchors FtsZ to the membrane together with SepF (Bi and Lutkenhaus, 1991; Jensen et al., 2005; Duman et al., 2013; Gola et al., 2015; Gupta et al., 2015), and ZapA that promotes higher order assemblies of FtsZ protofilaments (Gueiros-Filho and Losick, 2002). In the Gram-positive bacterium *Bacillus subtilis*, the membrane protein EzrA is also one of the earliest proteins that is recruited to the Z-ring, and is involved in the regulation of FtsZ polymerization and the coordination of peptidoglycan synthesis of the division septum (Levin et al., 1999; Claessen et al., 2008). After a significant time delay, a second set of division proteins, often referred to as “late proteins,” is recruited to the Z-ring, including the membrane proteins PBP 2B and FtsW, which are proposed to be responsible for the synthesis of the septal cell wall (Gamba et al., 2009). PBP 2B is the transpeptidase that is involved in the synthesis of septal peptidoglycan (Daniel et al., 2000), and FtsW has been assumed to facilitate the transport of cytosolically synthesized Lipid II, the lipid-linked precursor for peptidoglycan synthesis, into the existing cell wall (Mohammadi et al., 2011), although there appear to be other transporters (Meeske et al., 2015). However, most of the available information about the role of FtsW is based on studies with the Gram-negative bacterium *Escherichia coli*, but it has not been shown if the same effects also apply to the *B. subtilis* homolog.

FtsW is essential in *E. coli* (Ikeda et al., 1989; Khattar et al., 1994, 1997; Boyle et al., 1997). It belongs to the so-called SEDS family, which comprises polypeptides involved in shape, elongation, division, and sporulation (Errington et al., 2003). These proteins have 10 transmembrane domains and their genes are frequently in close proximity to genes for class B transpeptidases (Ikeda et al., 1989; Errington et al., 2003). This suggested the involvement of FtsW in the transport of the peptidoglycan precursor lipid II, a hypothesis that was supported by a detailed biochemical study (Mohammadi et al., 2011). Aside of this transport role, FtsW may also have a structural role as it is required in *E. coli* for the recruitment to the division site of the FtsI, the essential cell division specific PBP (denoted PBP 2B in *B. subtilis*) (Wang et al., 1998; Mercer and Weiss, 2002). It has also been suggested that FtsW is involved in the stabilization of the Z-ring, since in one of the first depletion experiments carried out in *E. coli*, long filamentous cells with no Z-rings were observed (Boyle et al., 1997). However, in later studies only a two-fold reduction of early protein localization was reported (Mercer and Weiss, 2002). In *Streptomyces coelicolor*, disruption of *ftsW* or *ftsI* leads to an early block in Z-ring formation in the aerial hyphae, and spiral polymers of FtsZ are unable to reorganize into rings (Mistry et al., 2008). Interestingly, FtsW and FtsZ of *Mycobacterium tubercolosis* were shown to interact *in vitro* through their C-terminal tails, which are extended relative to the *E. coli* counterparts (Datta et al., 2002). *Mycobacteria* lack FtsA, therefore such interaction might provide the membrane anchor for the Z-ring, something that is supported by the existence of a trimeric complex involving FtsZ, FtsW, and PBP3 in *Mycobacteria* (Datta et al., 2006).

The *B. subtilis* gene *spoVE* is located in the equivalent chromosomal position to *ftsW* of *E. coli*. It encodes a SEDS protein that is specifically required for sporulation, but mutations in this gene do not affect normal growth (Henriques et al., 1992; Errington et al., 2003). However, based on sequence similarity, another potential FtsW homolog is encoded by *ylaO* (Henriques et al., 1998), and is known to be essential (Kobayashi et al., 2003). Previous studies showed that the gene is necessary for septation (Suel et al., 2007) and that a GFP tagged version of YlaO assembles to the cell division site (Gamba et al., 2009), consequently this gene has often been referred to as being the homolog of *E. coli* FtsW in *B. subtilis*.

In this study, we set out to analyze the role of FtsW in the assembly of the *B. subtilis* divisome. We confirm that depletion of this protein results in a block of cell division and we show that the localization of the late cell division proteins FtsL and PBP 2B does not occur in the absence of FtsW, but also that FtsW's localization is dependent upon the late division proteins. We observed that upon prolonged absence of late division proteins, the Z-ring disassembles and, interestingly, we could restore Z-ring formation and constriction by re-inducing FtsW, which suggests that the stability of the Z-ring is stimulated by the binding of the late division proteins.

## Materials and methods

### Bacterial strains and growth conditions

Strains and plasmids used in this study are listed in Table 1.

**Table 1:** **Strains and plasmids used in this study**.

**Strain**	**Relevant features or genotype**	**Construction, source, or reference**
***B. subtilis***		
168	*trpC2*	Laboratory stock
2012	*amyE*::*spc* (*P_*xyl*_-gfpmut1-ftsL*)	Sievers and Errington, 2000
2020	*amyE*::*(P*_*xyl*_-*gfpmut1-ftsZ, spc)*	Gamba et al., 2009
2595J	*wprA::kan, epr::tet, yllB*::*pMUTIN4 (yllB′*::*lacZ lacI bla erm P_*spac*_-yllB-ylxA-ftsL-pbpB), amyE*::*P_*xyl*_-lytE-gfp*	Laboratory stock
3122	*pbpB*::*pSG5061, (cat P_*xyl*_-gfp-pbpB)*	Scheffers et al., 2004
3312	*ezrA-gfp cat*	Gamba et al., 2009
4731	*ftsW*::*pSG1151-ftsW-gfp cat*	Scowcroft, unpublished
RD158	*P_*spac*_-ftsW kan*	Daniel, unpublished
RD804	*ftsL799 (lacI aph-A3 P_*spac*_-pbpB)*, φ105J506 *(cat P_*xyl*_-ftsL)*	Daniel et al., 1998
PG77	*ftsW*::*pSG1151-ftsW-GFP cat*::*spc*	pCm::Spc → 4731
PG79	*P_*spac*_-ftsW kan, amyE*::*P_*xyl*_-gfp-ftsL spc*	2012 DNA → RD158
PG80	*P_*spac*_-ftsW kan, amyE*::*P_*xyl*_-gfp-ftsZ spc*	2020 DNA → RD158
PG82	*P_*spac*_-ftsW kan, ezrA-gfp cat*	3312 DNA → RD158
PG87	*P_*spac*_-ftsW kan, pbpB*::*P_*xyl*_-gfp-pbpB cat*	3122 DNA → RD158
PG92	*ftsL*::*lacI aph-A3 P_*spac*_-pbpB, φ105J506 (cat P_*xyl*_-ftsL), ftsW*::*pSG1151-ftsW-GFP cat*::*spc*	PG77 DNA → RD804
PG114	*amyE*::*P_*xyl*_-gfp-ftsZ spc, yllB*::*pMutin4 (P_*spac*_-yllB-ylxA-ftsL-pbpB)*	2595J DNA → 2020
**Plasmids**	**Relevant features or genotype**	**Construction, source, or reference**
pCm::Spc	*cat*::*spc*	Steinmetz and Richter, 1994
pSG441	*aph-A3 lacI Pspac*	Illing and Errington, 1991
pSG1151	*cat ‘gfp*	Feucht and Lewis, 2001
pSG1151-FtsW-gfp	*cat ‘ftsW-gfp*	H. Scowcroft, project thesis, Oxford University, 2000
pRD158	*pSG441 RBS—ftsW’*	This work

*Unless otherwise stated, all strains were made in 168 background. Genes responsible for resistance to antibiotics are abbreviated as follows: bla, ampicillin; cat, chloramphenicol; erm, erythromycin; phleo, phleomycin; kan, kanamycin; spc, spectinomycin; tet, tetracycline*.

*B. subtilis* strains were grown at 37°C in competence medium (Hamoen et al., 2002). When required, xylose and IPTG were used as inducers at concentrations of 0.2–0.5% and 1 mM respectively. Selection of transformants and short term maintenance of *B. subtilis* strains were performed on nutrient agar (Oxoid), supplemented when required with 10 μg/ml tetracycline, 5 μg/ml chloramphenicol, 50 μg/ml spectinomycin, 5 μg/ml kanamycin, 1 μg/ml phleomycin, or 0.5 μg/ml erythromycin with 25 μg/ml lincomycin.

### Construction of plasmids and bacterial strains

Plasmid pRD158 was constructed by inserting a PCR derived fragment of *ftsW* (also named *ylaO*) into plasmid pSG441, and carries a *P*_*spac*_ promoter located immediately in front of the RBS of the FtsW coding sequence. The *ftsW* fragment was amplified with the oligonucleotides W1 (5′-ATATCCTTCCCCTGTACAC-3′) and W2 (5′-ATATCCTTCCCCTGTACAC-3′), digested with *Xba*I and *Eco*RV and ligated to plasmid pSG441 previously digested with *Xba*I and *Sph*I and blunted using Klenow treatment.

Strain RD158 was then generated by Campbell integration of pRD158 into strain 168, selecting for kanamycin resistance in the presence of IPTG. The resulting transfomants were screened to confirm that they were dependent upon IPTG for viability and the location of the plasmid integration confirmed using PCR across the site of integration. One such transformant that exhibited the correct phenotype and had the correct genetic construction was then designated RD158.

Strain 4371 was generated by the integration into strain 168 of plasmid pSG1151-FtsW-gfp carrying a 3′-fragment of *ftsW* fused to the *gfp* gene. Campbell integration of the plasmid resulted in the expression of GFP fused to the C-terminus of FtsW under the native promoter. The correct integration of this plasmid was confirmed by PCR amplification of the site of insertion and sequencing across the fusion to ensure that the coding sequence of FtsW and GFP were joined as expected.

### Depletion of division proteins in vegetative cells

Overnight cultures grown at 37°C in competence medium were diluted 80-fold in the same medium supplemented with the appropriate inducers and grown at 37°C until they reached an O.D._600_ of ~0.3. Cells were then diluted 80 times in pre-warmed competence medium, in the presence or in the absence of the inducer. Incubation was continued at 37°C until cultures reached an O.D._600_ of ~0.3 (about 4 h), at which point cultures were re-diluted (40-fold) in the same pre-warmed medium.

### Sporulation and germination conditions

*B*. *subtilis* spores were prepared and germinated as previously described (Gamba et al., 2009). Briefly, strains were grown at 37°C in liquid sporulation medium until they reached an O.D._600_ of ~0.6 and then plated on sporulation agar plates after four serial five-fold dilutions. Plates were incubated at 30°C for 5–7 days. Spores were then scraped off the plates, washed in sterile water, and suspended in TE buffer (10 mM Tris-Cl pH 8.0, 1 mM EDTA) supplemented with 1.5 mg/ml lysozyme and incubated at 37°C for 1 h with shaking. Sodium dodecyl sulfate (SDS) was added to a final concentration of 5%, and the suspensions were incubated at 37°C for 30 min. Spores were then collected by centrifugation and washed four times with sterile water prior to being stored in water at 4°C. Spore preparations were subsequently washed twice a day for a week to avoid spontaneous germination.

For germination experiments, spores were diluted in germination medium (Hamoen and Errington, 2003), heat shocked for 30 min at 70°C, and quickly cooled on ice. The spore mixtures were then diluted in germination medium to an O.D._600_ of 0.3 in the presence or absence of the appropriate inducers, and incubated at 37°C with continuous shaking. Samples were collected for microscopy 3, 4, or 5 h after the beginning of the heat shock treatment (*t* = 0).

### Microscopic imaging

Samples were mounted on microscope slides coated with a thin layer of 1.2% agarose. Images were acquired with a Zeiss Axiovert 200M or a Zeiss Axiovert 135 microscope coupled to a Sony Cool-Snap HQ2 cooled CCD camera (Roper Scientific) and using Metamorph imaging software (Universal Imaging), or with a Deltavision microscope (Applied Precision) using a CoolSNAP HQ camera (Princeton Instruments) and softWorx imaging software (Applied Precision).

### Western blotting

Spores of strain PG87 were germinated in the presence of 0.5% xylose, and in the presence or absence of 1 mM IPTG. Optical density was measured at regular intervals and samples were collected, spun down and flash frozen in liquid nitrogen 3, 4, or 5 h after the beginning of the heat shock (8, 4, and 4 ml volumes respectively). Cell pellets were suspended in 100 μl of 1 × NuPAGE LDS Sample Buffer (Invitrogen) supplemented with Complete mini protease inhibitor (Roche), and broken by sonication (3 × 10 s pulses at 20% amplitude). Samples were denatured at 70°C for 10 min and loaded onto an SDS-PAGE gel (NuPAGE Bis-Tris 4–12% gradient gels, run in MOPS buffer, ThermoFisher Scientific), such that the amount of sample loaded was normalized relative to the optical density (O.D_.600_) measured at the time of collection. This allowed the comparison, at each time point, of the uninduced and induced sample, but not of samples taken at different time points, due to the different contribution that hydrated spores and spore coats gave to the total optical density. The resolved proteins were transferred onto a PVDF membrane (GE Healthcare) by using a wet procedure and Western blotting was performed according to standard methods. For the detection of PBP 2B, a 1:10,000 dilution of rabbit polyclonal anti-PBP2B serum was used. Anti-rabbit horseradish peroxidase-linked antiserum (Sigma) was used as secondary antibody at a dilution of 1:10,000. Protein bands were detected using an ImageQuant LAS 4000 mini digital imaging system (GE Healthcare). PVDF membranes were Coomassie stained after imaging to check for even protein transfer between induced and uninduced samples.

## Results

### Assembly of Z-rings after FtsW depletion

**Figure 1:**
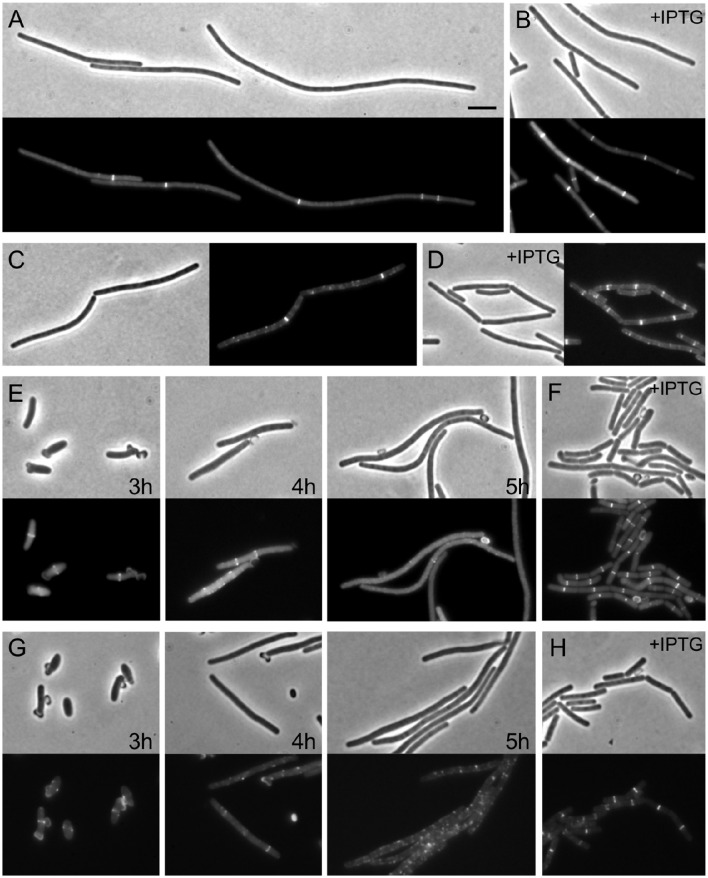
**Assembly of Z-rings after depletion of FtsW. (A,B)** Localization of FtsZ in FtsW depleted cells. Strain PG80 (*P_spac_-ftsW; amyE*::*Pxyl-gfp-ftsZ*) was grown in competence medium at 37°C in the absence **(A)** or in the presence **(B)** of 1 mM IPTG. Depletion of FtsW was achieved by diluting cells in warm medium in the absence of the inducer. Phase contrast and GFP images were taken after 8 h of depletion (~14 generations). **(C,D)** Localization of EzrA in FtsW depleted cells. Strain PG82 (*P_spac_-ftsW; ezrA-gfp*) was grown in competence medium at 37°C in the absence **(C)** or in the presence **(D)** of 1 mM IPTG. Depletion of FtsW was achieved by diluting cells in warm medium in the absence of the inducer. Phase contrast and GFP images were taken after 6 h of depletion (~10 generations). **(E–H)** Localization of FtsZ and EzrA in FtsW depleted germinating spores. Spores of strain PG80 **(E)** or PG82 **(G)** were heat shocked as described and germinated at 37°C in the absence of IPTG. Phase contrast and GFP images were taken after 3, 4, and 5 h, respectively. **(F–H)** Spores of strain PG80 **(F)** or PG82 **(H)** after 5 h of germination in the presence of 1 mM IPTG. Scale bar: 5 μm.

We constructed a strain in which FtsW could be depleted by placing the gene under the control of the IPTG inducible promoter *P*_*spac*_ (strain RD158). Microscopic analysis of depleted cells showed very long filaments without normal division septa (Figure 1), confirming that the gene is essential and is involved in cell division, as previously shown (Kobayashi et al., 2003; Suel et al., 2007).

Depletion of FtsW has been shown to have a strong effect on Z-ring stability in the Gram-positive organisms *S. coelicolor* and *Mycobacterium tuberculosis* (Boyle et al., 1997; Daniel et al., 1998; Henriques et al., 1998), and to test whether this was also the case in *B. subtilis*, the *P_spac_-ftsW* construct was introduced into a strain expressing *gfp* fused to *ftsZ* (PG80, *P_spac_-ftsW kan amyE*::*P_xyl_-gfp-ftsZ spc*). This strain was grown in a minimal medium supplemented with 0.2% xylose to induce expression and allow Z-rings to be seen. Growth of the strain in the absence of IPTG was found to cause a cell division defect detectable after about 90–120 min (about 3–4 generations) of incubation (although a significant degree of heterogeneity was observed in the cell population). After prolonged depletion of FtsW (4–6 h, 7–10 generations), long filaments were clearly visible while DAPI staining still showed a normal localization pattern of the nucleoids (not shown). Under these conditions, some bright Z-rings were visible, but only one or two rings per filament (Figure 1A). As the GFP-FtsZ fusion is not active, we also tested the effect of FtsW depletion using a strain containing an EzrA-GFP fusion that is known to be biologically active (Daniel, unpublished). Again filamentous cells with only one or sometimes two fluorescent bands were observed (Figure 1C). Thus, it seems likely that the Z-rings/EzrA observed in these filamentous cells are the remains of the last divisome that was active prior to FtsW becoming limiting. To confirm this, we made use of germinating spores. The dehydrated kernel of *B. subtilis* spore is devoid of any cell division structure and therefore the morphogenesis of spores into vegetative cells is not influenced by a previous cell division event. When spores were germinated in the absence of IPTG, and thus without *ftsW* expression, one or two GFP-FtsZ or EzrA-GFP rings became visible (Figures 1E,G), but after prolonged growth in the absence of IPTG, the rings disappeared (Figures 1E,G). Control experiments carried out in the presence of IPTG permitted the completion of at least 2 to 3 rounds of division, and Z-ring were clearly localized in all cells at the end of the experiment (Figures 1F,H). To compare the localization pattern of FtsZ upon depletion of other late proteins, spores of strain PG80 (*P_spac_-ftsW kan amyE*::*P_xyl_-gfp-ftsZ spc*) where germinated in parallel with spores of strain PG114 (*amyE*::*P_xyl_-gfp-ftsZ, P_spac_-yllB-ylxA-ftsL-pbpB*), which allowed the depletion of the FtsL and PBP 2B. Here again Z-ring formation could be observed at the early stages of depletion, as was seen with *ftsW*, but 5 h after germination (sufficient time for ~3 cell cycles under normal conditions) all rings were disassembled (Figure S1). This shows that the stability of the Z-rings is similarly affected by the absence of any of the late assembling division proteins.

### Z-ring reassembly upon FtsW re-induction

**Figure 2:**
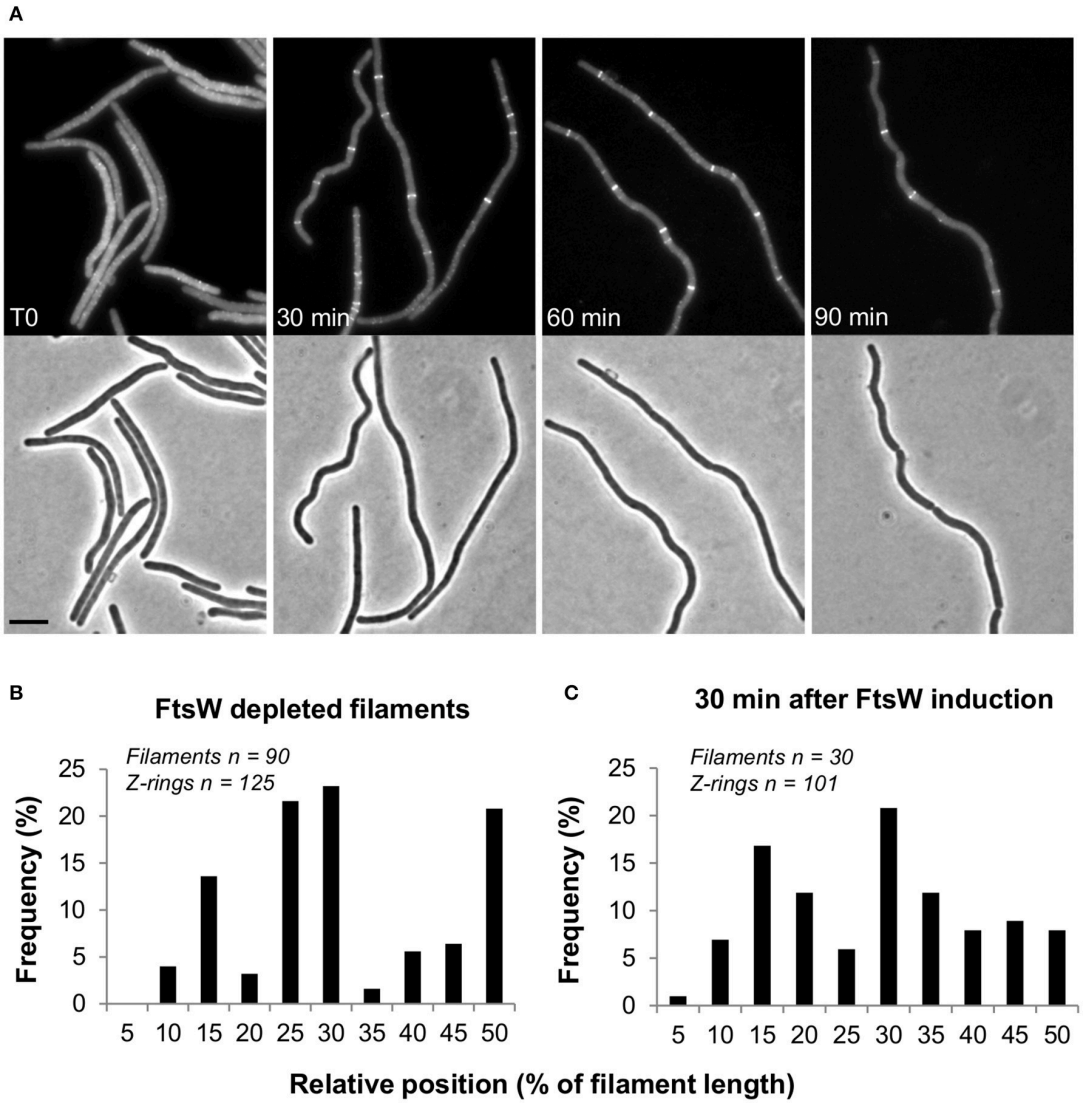
**Z-ring reassembly upon FtsW reinduction**. Spores of strain PG80 (*P_spac_-ftsW; amyE*::*P_xyl_-gfp-ftsZ*) were heat shocked as described and germinated at 37°C in the absence of IPTG. FtsW was reinduced by adding 1 mM IPTG 5 h after the beginning of germination. **(A)** Phase contrast and GFP images taken every 30 min after re-induction. Scale bar indicates 5 μm. **(B,C)** Analysis of the relative positions of Z-rings in FtsW depleted filaments **(B)** and 30 min after the re-induction of *ftsW* expression **(C)**, determined using a combination of phase and fluorescence images. In both cases, the positions of the detected Z-rings have been expressed as the distance from the nearest cell pole and as a percentage of the cell's total length.

In germinating spores Z-rings disassembled after prolonged depletion of FtsW or PBP2B/FtsL. In these experiments this would correspond to the time needed for germination (~3 h) and then 2–3 cell divisions (~35 min per cell cycle), in all about 5 h incubation. However, when *ftsW* expression was re-induced by adding 1 mM IPTG to the cultures, FtsZ rings re-formed along the filaments. Multiple Z-rings per filament were visible 30 min after re-induction, although the spacing between them was rather irregular (Figures 2B,C). These rings eventually constricted and sites of septation could be detected 1 h after the addition of IPTG (Figure 2A). Similar results were also obtained using EzrA-GFP as a marker of the Z-ring formation (not shown).

### Localization of PBP 2B, FtsL, and FtsW is interdependent

**Figure 3:**
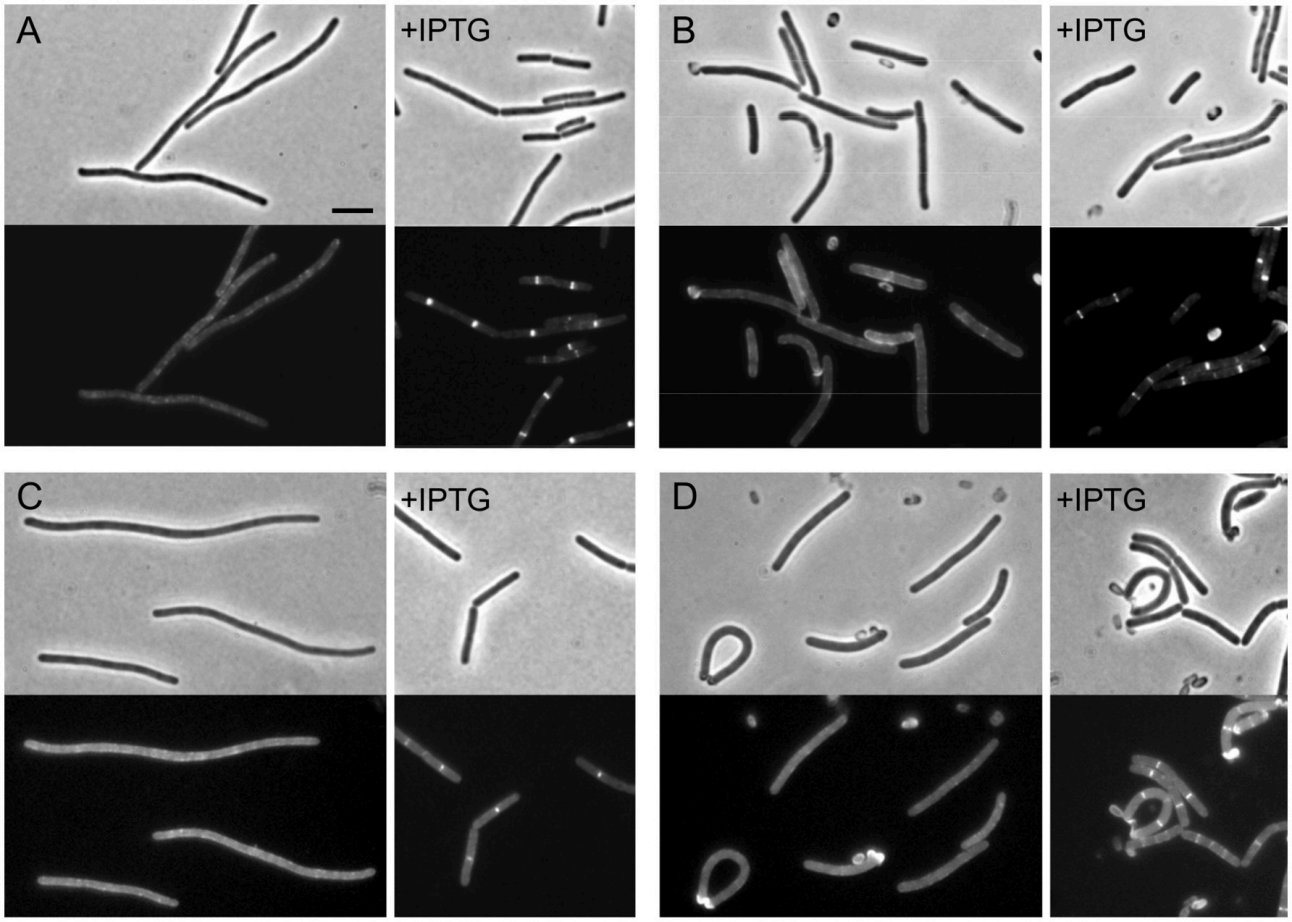
**The localization of PBP 2B and FtsL depend on FtsW. (A,B)** Localization of PBP 2B in FtsW depleted cells and germinating spores. **(A)** Strain PG87 (*P_spac_-ftsW; P_xyl_-gfp-pbpB*) was grown at 37°C in competence medium in the presence of 0.5% xylose and in the absence or in the presence of 1 mM IPTG. Phase contrast and GFP images were taken 7 h after depletion (~12 generations). **(B)** Spores of strain PG87 were heat shocked and germinated as described in the presence of 0.5% xylose and in the absence or in the presence of 0.5% xylose. Phase contrast and GFP images were taken 4 h after the beginning of germination. **(C,D)** Localization of FtsL in FtsW depleted cells and germinating spores. **(C)** Strain PG79 (*P_spac_-ftsW; P_xyl_-gfp-ftsL*) was grown at 37°C in competence medium in the presence of 0.5% xylose and in the absence or in the presence of 1 mM IPTG. Phase contrast and GFP images were taken 7 h after depletion (~12 generations). **(D)** Spores of strain PG79 were heat shocked and germinated as described in the presence of 0.5% xylose and in the absence of 0.5% xylose. Phase contrast and GFP images were taken 4 h after the beginning of germination. Scale bar indicates 5 μm.

In *E. coli*, recruitment of FtsI depends on FtsW. To test whether this is also the case in *B. subtilis*, the *P_spac_-ftsW* construct was integrated into strains carrying *gfp* fusions to *ftsL* and *pbpB*, generating strains PG79 and PG87 respectively. Depletion of FtsW was carried out in vegetative cells and germinating spores. In vegetative cells, depletion of FtsW was accompanied by disappearance of GFP signal for both FtsL and PBP 2B (Figures 3A,C). However, this could also be caused by the reduced frequency of Z-ring localization that we have shown before. We therefore repeated the experiments in germinating spores and confirmed that the absence of IPTG did not allow the formation of GFP bands, indicating that, when FtsW was depleted, PBP 2B and FtsL localization was lost (Figures 3B,D). As FtsL is known to be unstable when division is perturbed (Daniel and Errington, 2000) we focused on the stability of PBP 2B and used Western blotting to show that depletion of FtsW did not result in the degradation of PBP 2B (Figure S2) and hence the loss of localisation was not due to degradation of the GFP fusion.

**Figure 4:**
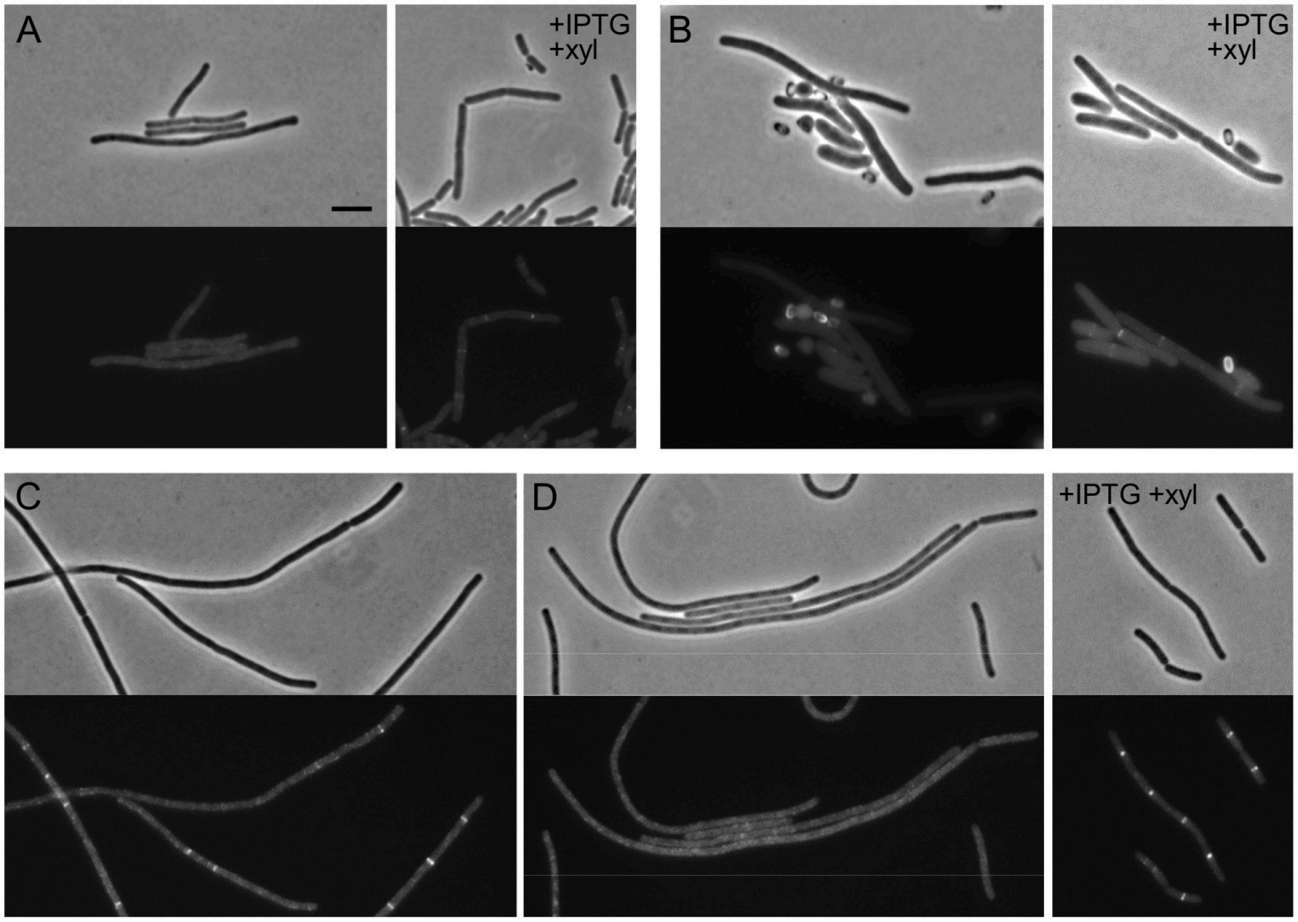
**The localization of FtsW, PBP 2B, and FtsL are interdependent. (A,B)** Localization of FtsW in PBP 2B depleted cells and germinating spores. **(A)** Strain PG92 (*P_spac_-pbpB; P_xyl_-ftsL, ftsW-gfp*) was grown at 37°C in competence medium in the presence of 0.5% xylose and in the absence or in the presence of 1 mM IPTG. Phase contrast and GFP images were taken 5 h after depletion (8–9 generations). **(B)** Spores of strain PG92 were heat shocked and germinated as described in the presence of 0.5% xylose and in the absence or in the presence of 0.5% xylose. Phase contrast and GFP images were taken 4 h after the beginning of germination. **(C,D)** Localization of FtsW in FtsL depleted cells. Strain PG92 was grown in competence medium at 37°C in the presence of 1 mM IPTG and in the absence or in the presence of 0.5% xylose. Phase contrast and GFP images were taken after 4 h **(C)** or 5 h **(D)** of depletion. Scale bar indicates 5 μm.

While the assembly of late division proteins follows multiple successive steps in *E. coli*, the recruitment of late proteins in *B. subtilis* seems to be a strongly cooperative process, with the late proteins PBP 2B, FtsL, DivIC, and DivIB being interdependent for localization (Errington et al., 2003). To study the hierarchy of recruitment of FtsW to the division site, a C-terminal GFP fusion to FtsW in its native locus (4731, *ftsW-gfp*) was introduced into a strain which allows the depletion of either PBP 2B or FtsL (Daniel et al., 1998), generating strain PG92. Strain PG92 was subsequently depleted for PBP 2B, by transferring it to a medium lacking IPTG. Cells became filamentous and the clear localization of FtsW-GFP at cell division sites disappeared (Figure 4A). More convincingly, no clear localization could be observed in germinating spores (Figure 4B). Strain PG92 allows also for the depletion of FtsL because *ftsL* is under the control of a xylose inducible promoter. While long filaments with no fluorescent signal could be detected in the absence of xylose (Figures 4C,D), this experiment could not be confirmed in germinating spores since the *P_xyl_* promoter is leaky during germination (not shown), and therefore efficient depletion is not possible. However, since the localization of FtsW depends on PBP 2B, it is expected that it will also depend on the presence of FtsL. We conclude that the localization of PBP 2B, FtsL and FtsW are interdependent in *B. subtilis*.

## Discussion

In this study, we analyzed the recruitment of FtsW to the division site and, in contrast to early immunofluorescence studies on other late proteins (Daniel and Errington, 2000; Daniel et al., 2000; Katis et al., 2000), we took advantage of the use of GFP fusions and spore germination.

When we looked at the localization of FtsZ or EzrA upon FtsW or PBP2B/FtsL depletion in vegetative cells or germinating spores, we observed only a few rings per filament, as if no new FtsZ ring could be formed (Figure 1, Figure S1). Nonetheless, reinduction of FtsW promoted the reassembly of Z-rings (Figure 2). Such reassembly did not occur regularly along the filament and it is possible that only partially unassembled Z-rings could reform and constrict. However, our data suggest that late division proteins are important for the stabilization of the Z-ring. Interestingly, a similar role was proposed in *E. coli* for the late division protein FtsN (Rico et al., 2010), while in *B. subtilis* FtsW, FtsL and PBP 2B appear to be equally important for this function.

The localization of FtsW is interdependent with the presence of late proteins PBP 2B and FtsL (Figures 3, 4). This is in agreement with the current cooperative model of divisome assembly in *B. subtilis* (Errington et al., 2003). Interestingly, in *E. coli* FtsW does not require the PBP 2B homolog, FtsI, to localize to the division site (Mercer and Weiss, 2002), while in *B. subtilis* FtsW localization is lost in the absence of the transpetidase PBP 2B (Figure 4). PBP 2B does not appear to be subjected to proteolytic degradation as FtsL and DivIC (Daniel et al., 2006). It remains unclear whether the absence of FtsW affects the stability of other late proteins, and if FtsW levels are also controlled by degradation.

## Author contributions

PG performed most experiments and prepared data, figures, and drafted text. RD and LH obtained funding, directed research, and edited text.

### Conflict of interest statement

The authors declare that the research was conducted in the absence of any commercial or financial relationships that could be construed as a potential conflict of interest.

## Supplementary material

Figure S1 **Comparison of FtsW and PBP 2B/FtsL depleted germinating spores**. Spores of strain PG80 (*P_spac_-ftsW, amyE*::*Pxyl-gfp-ftsZ*) **(A)** and of strain PG114 (*P_spac_-yllB-ylxA-ftsL-pbpB, amyE*::*P_xyl_-gfp-ftsZ*) **(B)** were germinated at 37°C in germination medium, in the absence of IPTG and in the presence of 0.25% xylose. Phase contrast and GFP images were taken 5 h after the beginning of the heat shock. Scale bar indicates 5 μm.

Figure S2 **Stability of PBP 2B upon depletion of FtsW**. Spores of strain PG87 (*P_spac_-ftsW, P_xyl_-gfp-pbpB*) were germinated at 37°C in the presence of 0.5% xylose and in the presence or absence of IPTG 1 mM. **(A)** Optical density measured at 600 nm, arrows indicate time points at which samples were collected. **(B)** Western blot analysis of PBP 2B levels in samples collected 3, 4, or 5h after the beginning of germination.
