# Supplementary figures and images for: Cooperative Recruitment of FtsW to the Division Site of Bacillus subtilis
Source: Front Microbiol. 2016 Nov 15;7:1808. doi: 10.3389/fmicb.2016.01808 (PMC5108771; doi:10.3389/fmicb.2016.01808)

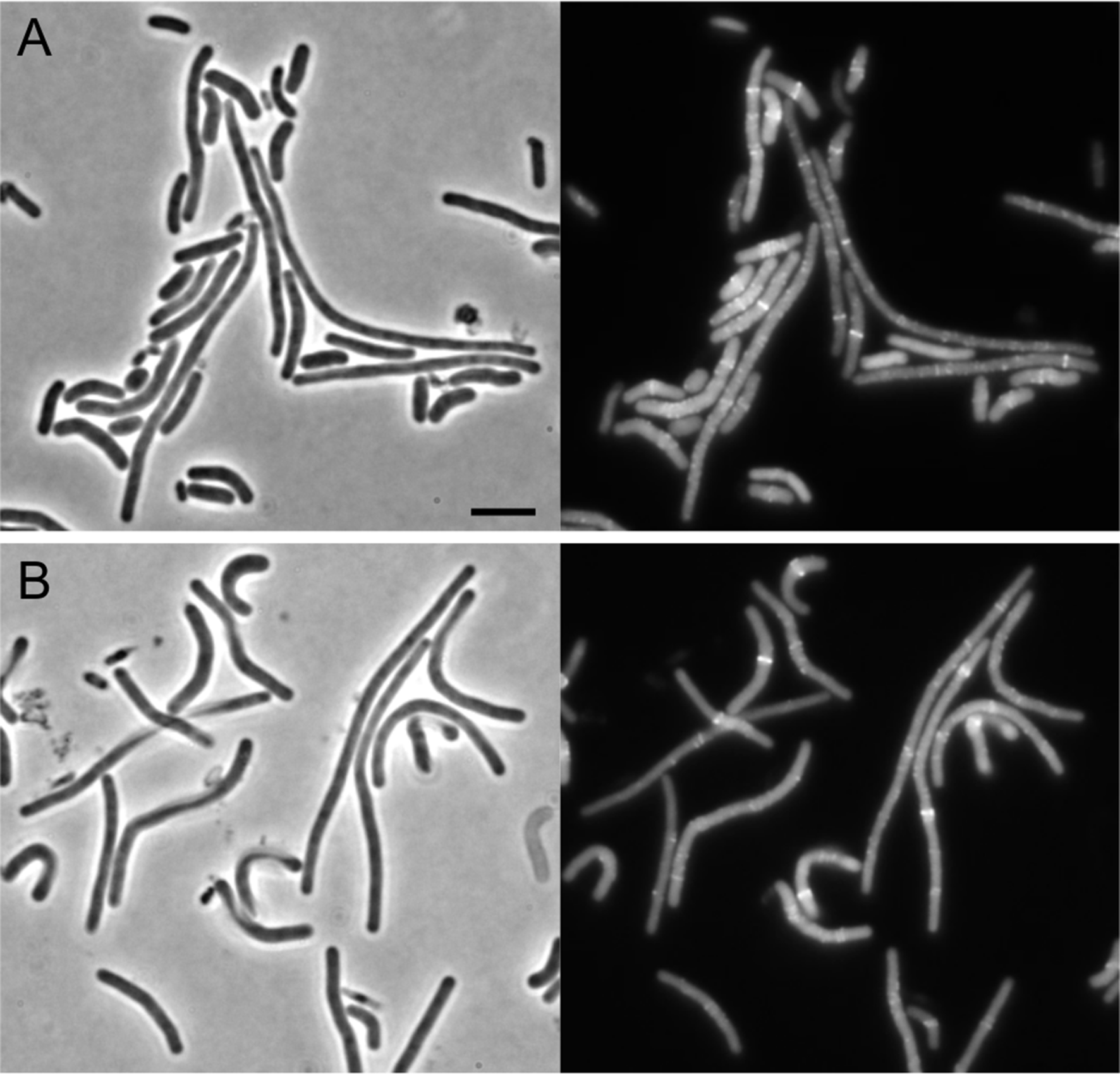

Supplement: Figure S1 — Comparison of FtsW and PBP 2B/FtsL depleted germinating spores. Spores of strain PG80 (Pspac-ftsW, amyE::Pxyl-gfp-ftsZ) (A) and of strain PG114 (Pspac-yllB-ylxA-ftsL-pbpB, amyE::Pxyl-gfp-ftsZ) (B) were germinated at 37°C in germination medium, in the absence of IPTG and in the presence of 0.25% xylose. Phase contrast and GFP images were taken 5 h after the beginning of the heat shock. Scale bar indicates 5 μm. [file Image1.TIFF]

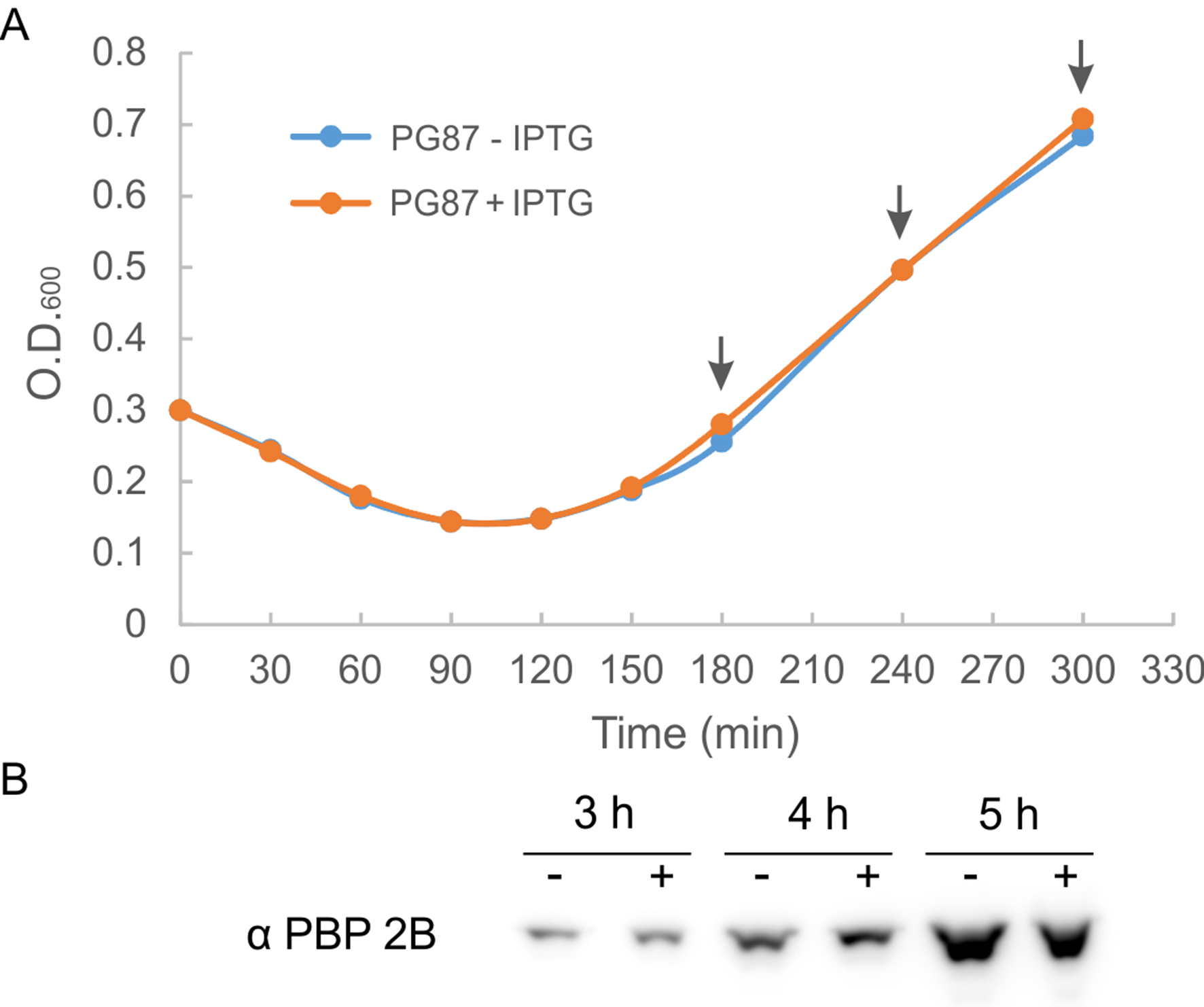

Supplement: Figure S2 — Stability of PBP 2B upon depletion of FtsW. Spores of strain PG87 (Pspac-ftsW, Pxyl-gfp-pbpB) were germinated at 37°C in the presence of 0.5% xylose and in the presence or absence of IPTG 1 mM. (A) Optical density measured at 600 nm, arrows indicate time points at which samples were collected. (B) Western blot analysis of PBP 2B levels in samples collected 3, 4, or 5h after the beginning of germination. [file Image2.TIF]
